# Meta-Analysis of Cytotoxicity Studies Using Machine Learning Models on Physical Properties of Plant Extract-Derived Silver Nanoparticles

**DOI:** 10.3390/ijms24044220

**Published:** 2023-02-20

**Authors:** Anjana S. Desai, Aparna Ashok, Zehra Edis, Samir Haj Bloukh, Mayur Gaikwad, Rajendra Patil, Brajesh Pandey, Neeru Bhagat

**Affiliations:** 1 Department of Applied Science, Symbiosis Institute of Technology, Symbiosis International (Deemed) University, Pune 412115, India; 2 Department of Pharmaceutical Sciences, College of Pharmacy and Health Sciences, Ajman University, Ajman P.O. Box 346, United Arab Emirates; 3 Center of Medical and Bio-allied Health Sciences Research, Ajman University, Ajman P.O. Box 346, United Arab Emirates; 4 Department of Clinical Sciences, College of Pharmacy and Health Science, Ajman University, Ajman P.O. Box 346, United Arab Emirates; 5 Department of Computer Sciences, Symbiosis Institute of Technology, Symbiosis International (Deemed) University, Pune 412115, India; 6 Department of Biotechnology, Savitribai Phule Pune University, Pune 411007, India

**Keywords:** Ag-NPs, cytotoxicity, HEK293, PC 12 cell line, machine learning, Decision Tree, Random Forest, k-means clustering, regression metrics

## Abstract

Silver nanoparticles (Ag-NPs) demonstrate unique properties and their use is exponentially increasing in various applications. The potential impact of Ag-NPs on human health is debatable in terms of toxicity. The present study deals with MTT(3-(4, 5-dimethylthiazol-2-yl)-2, 5-diphenyl-tetrazolium-bromide) assay on Ag-NPs. We measured the cell activity resulting from molecules’ mitochondrial cleavage through a spectrophotometer. The machine learning models Decision Tree (DT) and Random Forest (RF) were utilized to comprehend the relationship between the physical parameters of NPs and their cytotoxicity. The input features used for the machine learning were reducing agent, types of cell lines, exposure time, particle size, hydrodynamic diameter, zeta potential, wavelength, concentration, and cell viability. These parameters were extracted from the literature, segregated, and developed into a dataset in terms of cell viability and concentration of NPs. DT helped in classifying the parameters by applying threshold conditions. The same conditions were applied to RF to extort the predictions. K-means clustering was used on the dataset for comparison. The performance of the models was evaluated through regression metrics, viz. root mean square error (RMSE) and R^2^. The obtained high value of R^2^ and low value of RMSE denote an accurate prediction that could best fit the dataset. DT performed better than RF in predicting the toxicity parameter. We suggest using algorithms for optimizing and designing the synthesis of Ag-NPs in extended applications such as drug delivery and cancer treatments.

## 1. Introduction

In the last few decades, nanomaterials (NMs) have been at the forefront of materials research for various applications. Due to their unique properties and designs compared to conventional bulk materials, they have been considered “materials of the 21st century” [1]. There are numerous avenues of application for nanoparticles, such as in the industrial sector, medical and biomedical fields, health care devices, engineering, electronics, and environmental studies [2]. There is a significant focus on synthesizing NMs as nanospheres, nanotubes, fullerenes, and quantum dots for various applications [3]. Ag-NPs are in high demand and are used extensively in consumer products such as cosmetics and ointments [4]. Ag-NPs are also used in medicine, therapeutic devices, pharmacology, biotechnology, electronics, engineering, energy, magnetic fields, and environmental remediation [5]. Besides these applications, Ag-NPs have gained importance in industrial sectors, including textiles, food, and consumer products, due to their inherent and effective antibacterial properties [6]. Ag-NPs are also being explored in healthcare, women’s hygiene products, paints, sunscreen, biosensors, clothing, and electronics [4].

A high surface-to-volume ratio of Ag-NPs enhances their antimicrobial properties, which makes them efficient in wound healing and topical drug administration [7]. Despite these benefits, Ag-NPs are also known to exhibit toxicity. The strong oxidative property of Ag-NPs results in the release of silver ions (Ag^+^). These silver ions interact with the cells and adversely affect the system causing cytotoxicity, genotoxicity, and immunological responses leading to cell death (apoptosis) [8,9,10,11]. Consequently, the use of Ag-NPs in biological systems is limited [7,12]. Thus, using Ag-NPs raises concerns about exposure in human or animal bodies due to their easy penetration into the tissues and reported toxicities [13]. Moreover, it is well known from the literature that Ag^+^ ions have shown enhanced toxicity compared to elemental Ag and nanoparticles [11]. Colloidal solutions of Ag products for medical purposes release Ag^+^ ions, which may directly affect human health [14]. The mechanism of cytotoxicity of Ag-NPs is not entirely known, and the exact interaction processes of NPs with biological entities are yet to be understood [15,16,17,18]. Several studies have revealed that varied characteristics of Ag-NPs (e.g., particle size, shape, the dose of NPs, time of exposure, and agglomeration of Ag-NPs) play a vital role in affecting cytotoxicity. Different studies have shown that the physical properties of NPs strongly depend upon their kinetic growth at each stage of synthesis [19,20]. Undergoing a comprehensive toxicity study through the conventional method is tedious and time-consuming. In the current consumerism-driven world, we require a faster method to predict the parameters that affect cytotoxicity; hence, machine learning could be an alternative [21]. It is imperative to establish the predictive relationships between cytotoxicity and the physical properties that play a crucial role in tuning the cytotoxic behavior of Ag-NPs. In 2019, Hagar I. Labouta et al. performed a meta-analysis on published data on the behavior of numerous organic and inorganic NPs towards cytotoxicity and used the classification-based Decision Tree models [21,22]. In 2020, Yi-Hsein Cheng et al. used 376 data sets from the literature and performed physiologically-based pharmacokinetic (PBPK) modelling and simulations. These models helped in predicting tumor delivery efficiency [23]. Recently, Lie Liu et al., 2021, reported a meta-analysis of the published data using DT and RF, and predicted the key variables affecting Ag-NPs-mediated cytotoxicity [21]. Nevertheless, the intrinsic physical properties acting as attributes/features in earlier-used modelling methods are inadequate to understand the complex behavior of nanoparticles with different cell lines. The list of nanoparticles considered in data mining may affect the predictive results of machine learning because different nanoparticles exhibit various physical properties. In the present study, we have used the previously reported Ag-NPs synthesized using turmeric extract considering nanoparticle toxicity issues as well [24,25,26,27]. This study presents the anti-cancer property of Ag-NPs using an MTT assay. The meta-analysis of Ag-NPs on normal cell lines and carcinoma cell lines was performed using supervised learning algorithms, i.e., Decision Tree (DT) and the Random Forest (RF), as well as unsupervised learning algorithm k-means clustering. To our knowledge, this is the first time that acomparative study of experimental analysis amalgamated with ML predictions has been presented.

## 2. Results

In our earlier reported work [24], the physical properties and stability of prepared Ag-NPs were characterized using XRD, XPS, FESEM, TEM, DLS, and Zeta potential techniques. Along with these studies, the optical properties of Ag-NPs were described using UV-Vis Spectroscopy.

### 2.1. Role of Optical Properties

**Figure 1:**
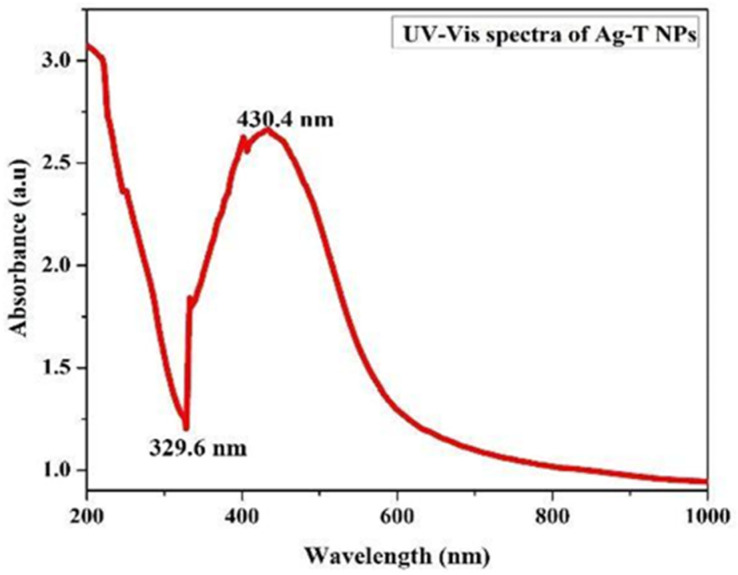
The UV-Vis absorption spectrum of Ag-T-NPs.

Preliminary investigations of Ag-T-NPs formation were carried out with an ultraviolet-visible (UV-Vis) spectroscopic analysis from 200 to 800 nm. The peak occurringat 430.4 nm for the Ag-T NPs is usually the characteristic surface plasmon resonance (SPR) peak for metal nanoparticles when their reactions are carried out in laboratory conditions. The UV–visible spectrum of the aqueous medium containing Ag-T-NPs showed an absorption peak around 430.4 nm, as shown in Figure 1.

Transverse oscillations of electrons are credited with causing these peaks, and light scattering also plays a role.

The trough at 329.6 nm in the spectrum depends upon the particle radius as a measure of the refractive index [28,29]. A shoulder at 400 nm shows an increase in absorbance. The precise nature of this shoulder is known to be a component of plasmon resonance, and theoretical simulations using Mie formulations can predict its occurrence. When the rate of silver nanoparticle creation has accelerated, and the particle size has grown, the increase in absorbance is evident with the position of the peak shifting toward longer wavelengths.

### 2.2. Effect of Ag-NPs Induced Cytotoxicity on PC 12 Cells

**Figure 2:**
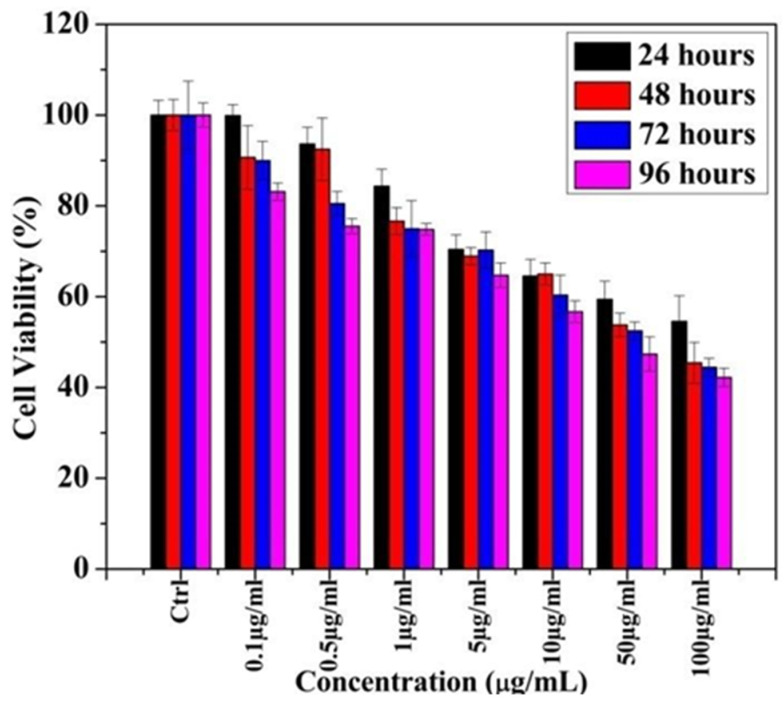
MTT assay of cell cytotoxicity in PC 12 cell lines using Ag-T- NPs.

Figure 2 shows that the increased concentration of Ag-T-NPs increases the toxicity on PC 12 (Pheochromocytoma) cell lines.

From the MTT assay, it was observed that, at 1 µg/mL, cell viability reduces appreciably after 24 hours (h) and remains unchanged for 72 and 96 h. At 24 h, cell viability decreases with an increased concentration of NPs. The same trend was observed for 48, 72, and 96 h of exposure time. Concentrations that were higher than 5 µg/mL showed a reduced cell viability compared toremaining concentrations. This finding suggested that cytotoxicity induced by the Ag-T-NPs is time- and concentration-dependent.

### 2.3. Significance of Physical Parameters on Cytotoxicity

#### 2.3.1. Role of the Selected Input Features on the Cytotoxicity

Ag-NPs show a cytotoxic nature, and the parameters that affect the cytotoxicity are particle size, capping agents, reducing agents, zeta potential, shape, chemical compositions, exposure time, dosage, and the wavelength of NPs.

#### 2.3.2. Effect of Particle Size

Particle size is reported to be a key parameter for deciding the cytotoxic nature of Ag-NPs [30]. It is evident from the literature that particle size affects the surface area-to-volume ratio and surface reactivity of nanoparticles [31,32,33]. One possible reason could be that the increased surface area of nanoparticles increases the number of surface atoms or molecules in an exponential trend, which offers high reactivity [34]. Also, different particle sizes show distinct interactions with differentcell lines [35]. It is evident from the research that, as the particle size decreases, the cytotoxicity increases. This statement can be validated by Carlson et al., who synthesized 15 nm and 55 nm hydrocarbon-coated Ag NPs. They observed that the generation of reactive oxygen species (ROS) is higherfor 15 nm as compared to 55 nm Ag NPS exposed on macrophage cell lines [31]. Similar results were found in the work of Liu et al., where 5 nm Ag-T-NPs proved to be more toxic than 20 and 50 nm Ag-NPs on four different cell lines (A549, HEPG2, MCF-7, and SGC-7901) [21]. One possible reason for this could be the easy internalization of smaller particle sizes into cell membranes.

#### 2.3.3. Effect of Capping Agent

The capping of an electrostatic layer is required to curtail the agglomeration and stabilize Ag-NPs. Capping agents help modify the surface chemistry of NPs by stabilizing them, offering a definite shape, and reducing the Ag^+^ ions. From the existing literature, it has been inferred that polysaccharide-coated Ag-NPs show suitable antimicrobial properties against eukaryotic cells with no toxicity [36]. A difference in toxicity was observed for coated (PVP and citrate coating) and uncoated Ag-NPs when used on J774A.1, a macrophage, and HT29 epithelial cells [37]. This observed difference in toxicity supports the claim that the capping agent plays a significant role in cytotoxicity. In 2014, Gliga et al. studied the size-dependent toxicity against BEAS-2B cells of commercially acquired 40 nm and 75 nm citrate-coated Ag-NPS, and 10 nm citrate and PVP-coated Ag-NPs, which were directly used [30]. From the obtained results, irrespective of coatings, more cytotoxicity was seen in 10 nm Ag NPs compared to 40 and 75 nm Ag-NPs. However, no difference in cytotoxicity was observed for 10 nm citrate and PVP-coated Ag-NPs. These findings suggest that the particle size is the critical parameter that triggers cytotoxicity, moreso than the capping agent.

#### 2.3.4. Effect of Biological Reducing Agents (Plant Extract)

The most popular ways of synthesizing Ag-NPs are chemical and biological routes. The biological reducing agents such as plant extracts form a natural capping on Ag NPs and inhibit the growth of particles [38]. In our previous study, we observed that biologically synthesized NPs showed lesser cytotoxicity compared to chemically synthesized NPs [24].

Ag-NPs show a cytotoxic nature, and the parameters that affect their cytotoxicity are particle size, capping agents, reducing agents, zeta potential, shape, chemical compositions, exposure time, dosage, and the wavelength of the NPs.

#### 2.3.5. Effect of Zeta Potential on Cytotoxicity

Colloidal stability plays a significant role in cytotoxicity. This stability can be achieved in two ways: steric stabilization or electrostatic stabilization. Steric stabilization is achieved through capping, and electrostatic stabilization is brought about by adding foreign charge species with charges opposite to the system. Due to the distribution of this opposite charge, particles do not agglomerate. It has been observed that nanoparticles with Zeta potential values greater than ±30 mV are typically considered to be stable [39]. Stability leads to a stronger interaction between positively charged nanoparticles and cell membranes [40]. It is reported that the nanoparticles with negative zeta potential cause less damage to the cells than those with positive zeta potential, maybe because the cell membranes are negatively charged [41,42,43]. One study found that the positively charged NPs showed more significant toxicity to HeLa and NIH/3T3 cells than negatively charged NPs. More interactions lead to easier internalization of NPs into the cell and, hence, trigger the toxicity, leading to damage and the arrest of cells in the Go/G1 phase; strong binding between the NPs and the negatively charged DNA could be a probable reason for this [44,45].

#### 2.3.6. Effect of Nanoparticles Morphology on Cytotoxicity

Depending on the synthesis techniques and the parameters, such as temperature, pH, reducing agent, and other experimental conditions, nanoparticles acquire different morphologies such as spheres, ellipsoids, pillars, sheets, cubes, rods, and many more. Numerous research papers have found that the shape of nanoparticles affects cytotoxicity [46,47,48,49]. For example, spherical nanoparticles are more subject to endocytosis (the process by which cells absorb external material by engulfing it with its membrane) than nanofibers and nanotubes [50]. Large portions of the cells are killed by plate-like and needle-like NPs compared to rod-shaped and spherical nanoparticles [51].

#### 2.3.7. Effect of Chemical Composition on Cytotoxicity

Apart from the size and shape of nanoparticles, other parameters trigger cytotoxicity, and one among them is the chemical composition. In the case of metal nanoparticles, the main reason for the toxic effect is the leakage of metal ions from the NPs while interacting with cells. This toxicity also depends on the chemical composition of the NPs. Higher concentrations of other metal ions, such as iron (Fe) and zinc (Zn), damage the cells despite their biological applications. While synthesizing the NPs, researchers attempted to reduce their cytotoxicity by coating the core of the NPs with a silica layer, with thick layers of polymer, and by using specific non-toxic protocols [41].

#### 2.3.8. Effect of Exposure Time on Cytotoxicity

The time duration of the exposure of cells to nanoparticles affects the cytotoxicity. In one study, the Ag-NPs were exposed for 6 and 12 h. It was clear that cytotoxicity increased in the case of NPs exposure for 12 h compared to those exposed for 6 h [52]. The possible reason for this could be the oxidation of nanoparticles to Ag^+^ ions (Trojan horse effect) that are inherently toxic. Thus, the continuous exposure time will increase the concentration of Ag^+^ ions in the solution, resulting in more significant cytotoxicity [53].

#### 2.3.9. Effect of Wavelength on Cytotoxicity

Researchers coated NPs with various substances to reduce toxicity. It has been observed that coating brings about a modification in the optical properties of NPs. The coating type and thickness modify the surface plasmonic resonance (SPR) of Ag-NPs, which is observed as a shift in peak position in UV-Vis spectral studies. Thus, coating substances such as organic, inorganic, and polymer can enhance the optical properties of Ag-NPs [54].

#### 2.3.10. Effect of Concentration on Cytotoxicity

The concentration or the dosage of nanoparticles is another critical parameter that affects toxicity. Although it is a known fact that toxicity depends upon the concentration, it is challenging to find the minimum concentration level of NPs that induces toxicity. The percentage of toxicity varies with the concentration of NPs as per the cell line [55].

## 3. Discussion

### 3.1. Machine Learning Models to Predict Cytotoxicity Influencing Parameters

Machine learning works well when the correlation of input features is optimized. The predicted outcome must agree with the conditions involved while performing the regression and classification analyses through Decision Tree (DT), Random Forest (RF), and clustering. A supervised learning technique follows specific steps before arriving at the prediction. Under supervised machine learning (ML), datasets are distributed as test and training datasets. First, algorithms are trained on the labelled dataset to match the outcome (in terms of cytotoxicity). Then, the model is validated on a test dataset, a subset of the training dataset. The input features are selected for optimal predicted output. Decision Tree (DT) and Random Forest (RF), from the Orange open-source toolkit, were used [56]. The data are collected and tailored into an m × n matrix, where m corresponds to 1135 datasets and n corresponds to the nine features. The information was collected on two datasets based on the cell types, i.e., normal cell lines and carcinoma cell lines. The DT and RF algorithms were applied to both datasets with the same input parameters. The input parameters were reducing agent, carcinoma and normal cell lines, exposure time, particle size, hydrodynamic diameter, zeta potential, wavelength, concentration, and cell viability.

Certain limitations were imposed on the normal cell line, including (i) cell viability less than or equal to 50% (which implies that the nanoparticles are toxic to the cells); (ii) cell viability greater than 50% (indicating that the nanoparticles are non-toxic to cells). The condition proposed for the carcinoma cell line was (i) cell viability less than or equal to 50%, implying that nanoparticles are toxic to the cells and hence favourable for us; (ii) cell viability greater than 50%, indicating that nanoparticles are non-toxic to cells and non-favourable in this case.

As an ensemble learning tool built upon DT, RF consists of multiple classifications and regression DTs. Each DT or random tree of RF model was grown in the following manner.

**Decision tree:** This supervised machine learning technique is used to solve classification problems. The classifier has a tree-structured nature, having nodes, branches, and leaves. The tree begins with a root node, extends its branches further, and constructs a tree-like structure until reaching the output. The internal nodes give the features of the dataset, a branch gives the decision rules, and the leaf node gives the outcome. The decision node and leaf node are the major nodes that play an important role in developing a Decision Tree. The first is the decision nodes having multiple branches, and the second is the leaf nodes. Decision nodes have multiple branches for making any kind of decision. Leaf nodes do not contain any further branches and give the output of the decisions made. The features of the given dataset are vital in performing the decisions and the tests to be conducted on the dataset. They are a graphical representation of the possible solutions to a decision or problem of interest based on the given conditions. A Decision Tree asks the question in such a way that the answer is Yes or No, which splits the tree further into sub-trees.

**Random forest:** The supervised learning techniques are combined with the calculations of many Decision Trees to obtain a final output result. RF mostly creates multiple Decision Trees and the outcomes of the trees are not correlated, as the features are selected without replacement, reducing the possibility of overfitting by averaging down the result. It is an ensemble of Decision Trees and, to develop many Decision Trees, the dataset must be divided into different subsets by randomly choosing the feature with which the data tree needs to be trained. The algorithm begins with the selection of random samples from a given dataset. An algorithm will be constructed for every sample consisting of one Decision Tree each, and then the prediction is obtained from each Decision Tree. For classification problems, the output is the class chosen by most trees. The most frequent categorical variable will yield the predicted class. In fact, Decision Trees consider all of the possible features to split to produce an outcome, whereas Random Forest selects only the subset of the features. Each tree in the ensemble has data taken from the training set. From the entire training set, one-third of the set is taken as test data. The determination of the prediction varies according to the statement of the problem.

The study leverages multiple hyperparameters in Random Forest and Decision Tree provided by Orange Tool. The result in Table 1 uses these hyperparameters. Multiple values of each hyperparameter were experimented on. The resultant table provides the best results. 

**Clustering:** Cluster denotes a group of similar items occurring as an assemblage. The technique follows the dividing data points into groups such that data points in the same groups are more similar to other data points in the same group and dissimilar to those in other groups. It is the collection of objects based on similarities and dissimilarities between them. 

**K-Means clustering:** The first step is to randomly select the number of clusters, each represented by a variable ‘k.’ Next, each cluster is assigned a centroid, i.e., the centre of that particular cluster. It is important to define the centroids as far off from each other as possible to reduce variation. After all of the centroids are specified, each data point is assigned to the cluster whose centroid is at the closest distance. Once all of the data points are assigned to respective clusters, the centroid is again assigned for each cluster. Once again, all of the data points are rearranged in a specific cluster based on their distance from the newly-defined centroids. This process is repeated until the centroids stop moving from their positions.

The original data are sampled repeatedly, and at each sampling, a set of features is randomly selected from each node pool, resulting in the best cart segmentation algorithm selection. Finally, a forest is grown by aggregating the random features (classifiers) and allowing each tree to determine the most likely classification. RF models are often more accurate and resilient than DT classifiers in the presence of noise and outliers.

#### 3.1.1. The Statistical Techniques for Prediction and Evaluation 

Sampling is conducted on the average of classes on randomly selected data points of the entire dataset. The importance of stratified sampling in cross-validation is to ensure that training and testing sets have a relative proportion of the features of interest compared to the original dataset. The accuracy of the predictions made by the generated Decision Tree (DT) and Random Forest (RF) model was evaluated. Usually, cross-validation is done to reduce the error produced by the inappropriate selection of target class variables. It also ensures that no data point is over or under-represented in training and test sets to obtain a more accurate performance estimate. The performance of an algorithm can be visualized using a specialized table structure called a confusion matrix. The confusion matrix is a performance measure of ML classification problems where the output can be two or more classes. It helps to measure Recall, Precision, Accuracy, and f1-score. Recall indicates a high possibility of correctly predicted values from the positive classes. Precision shows the values that turned out to be positive from the classes predicted as positive. It is difficult to compare two models with low Precision and high Recall. The f1-score is also used to identify the performance of algorithms. f1-score is the measure of the ability of a classifier to distinguish between classes. The probability curve plotted against the true and false positive rates at the threshold values when f1-score = 1 can distinguish the positive and negative class points perfectly. If f1-score = 0, all positive values are predicted as negative values and vice versa. An f1-score, such that 0.5 < f1-score < 1, indicates that the algorithm can detect more true positives and negatives than false positives and false negatives. A f1-score = 0.5 shows the inability to distinguish between the positive and negative class points.

#### 3.1.2. Estimating the Performance of the Models

Prediction accuracy was estimated for the two models that were developed, Decision Tree (DT) and Random Forest (RF), based on the 10-fold cross-validation method. The performance was evaluated in terms of Precision (PR), Recall (RE), Accuracy (AC), and f1-score. These values indicate the prediction competence of the generated models, where a higher value denotes a better model, and a value equivalent to 1 suggests a perfect model. If the accuracy value is >70%, it is considered that the classification model has a high prediction capability. The area under the receiver operating characteristic curve was evaluated for the classifier’s performance by plotting all combinations of decision threshold values in False and True Positive Rates (FPR and TPR, respectively).

#### 3.1.3. Validation of the Models and Their Comparison 

**Figure 3:**
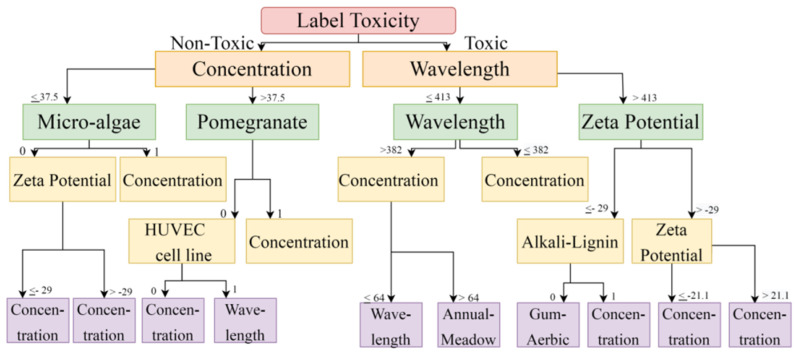
Toxicity is vital in predicting cell viability using a Decision Tree. When solutions are non-toxic, it depends upon concentration; when solutions are toxic, it depends upon wavelength.

**Figure 4:**
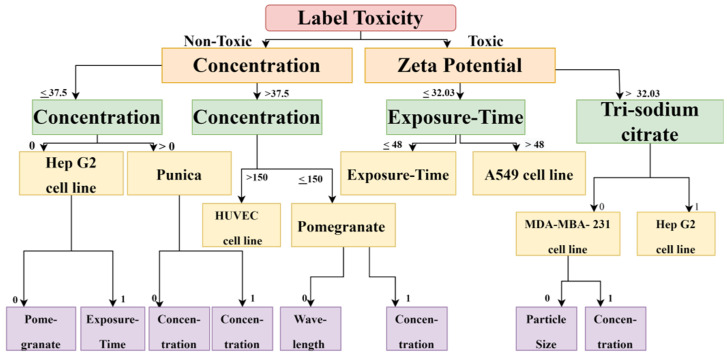
The decision Tree diagram confirms that, for calculating cytotoxicity and cell viability for carcinoma cell lines, zeta potential, exposure time, reducing agent, and concentration are important factors.

The DT and RF models demonstrated optimum accuracy through the nine input parameters, and conditions were imposed on the dataset for performing the CART (Classification and Regression Tree) algorithm. The prediction metrics were utilized to check the accuracy of our predictions and convey the numeral deviation from actual values. The mean square error (MSE), root mean square error (RMSE), mean absolute error (MAE), and R^2^ are all measures used to assess the model’s effectiveness in regression analysis. MSE is the average squared difference between the original and predicted values in the dataset. In contrast, MAE is the average of the absolute error difference between the actual and anticipated values in the dataset, i.e., assessing the variance of the residuals. RMSE measures the standard deviation of the residuals. R^2^ measures how well a regression model fits a dataset and how well it reproduces observed findings based on the proportion of total variation in outcomes that the model is responsible for explaining. Figure 3 and Figure 4 show the Decision Tree obtained according to the classification based on the cell lines and their toxicity.

Classification was performed to differentiate between normal and cancer cells based on the parameters provided. It was done to reflect that normal cells may have different feature values with different cell viability. The regression was performed to predict the cell viability for normal and cancer cells. Regression helps to analyze the different parameters that can affect cell viability. Thus, both the classification and regression helped to validate the cell viability based on different features. In regression analysis, MAE, MSE, RMSE, R squared, and Adjusted R^2^ metrics were mainly used to evaluate the model’s performance. The values are from the test results.

When regression analysis was performed on the Decision Tree and Random Forest model, the R^2^ (0.97) of the DT model was higher than the R^2^ (0.87) of the RF model, and the RMSE (4.22) value of DT was lower than the RMSE (9.75) value of RF. The high value of R^2^ and low value of RMSE indicate that the prediction is accurate, suggesting that the Decision Tree performed better than the Random Forest in predicting the toxicity parameter. The prediction was more precise and accurate, and best fit the dataset, as shown in Table 2 and Table 3. With 10-fold cross-validation, the Decision Tree and Random Forest models provided nearly the same results. Thus, both DT and RF are suitable for the toxicity classification of carcinoma and normal cell lines. 

Further, 10-fold cross-validation, Decision Tree and Random Forest provided nearly the same results. The 10-fold cross-validation was performed using Orange Tool, which returns only the best result. Therefore, multiple results are not provided.

The k-fold cross-validation (explained below) was used to create data splits. These data splits are in a ratio of 9:1, where 90% were training data and 10% were testing data. The total training data points were 734 and 81 testing for carcinoma. In addition, we used 450 data points as training and 50 rows of data as testing for normal cell lines.

The ratio between the total data of normal to carcinoma was 815:500, i.e., 8:5. Since the dataset has a higher number of data collected on the papers worked on carcinoma cell lines in comparison with normal cell lines, the ratio splitting preferred carcinoma over normal cell lines.

The test score’s accuracy, f1-score, Precision, and Recall confirm that DT and RF classify non-toxic and toxic parameters correctly, and DT performed better than RF in classifying them.

**K-fold cross-validation:** The data samples are chosen in the same proportion from a population based on the characteristics. The accuracy in the prediction of the generated Decision Tree (DT) and Random Forest (RF) model was evaluated. Sampling was conducted on the average of classes on the data points selected randomly from the entire dataset. The inclusion of stratified sampling is vital in cross-validation to ensure that training and testing sets have an equal proportion of the features of interest compared to the original dataset. Usually, cross-validation is done to reduce the error arising from the inappropriate selection of target class variables. It also ensures that no data point is over- or under-represented in the training and test sets to give a more accurate estimation of performance or error. 

The dataset was randomly divided into independent k subsets, where k = 10, and where the entire dataset kept a union between the subsets. The intersection between these subsets should be null and void. Then, k-1, i.e., nine subsets of the entire data, were taken for training to produce classifiers, and the existing subset was used for substantiation. The average value of k testing results is, finally, restored to the model. 

**Confusion matrix:** An algorithm’s performance, frequently that of a supervised learning algorithm, can be visualized using a specialized table structure called a confusion matrix, also known as an error matrix, in machine learning and, more specifically, in the problem of statistical classification. The confusion matrix is a performance measurement for ML classification problems where the output can be two or more classes.

The efficiency of a classification model is evaluated using an N × N matrix termed the confusion matrix, where N is the total number of target classes. The developed machine learning model compared the predicted target values to the actual target values in the matrix. The confusion matrix shows the prediction of toxic and non-toxic data (Table 4).

The dataset was trained and tested for the confusion matrix of normal cell lines with the implemented DT and RF algorithms. For both the training and test set, the DT and RF correctly classified all non-toxic data points as 0 and toxic data points as 1.

For the confusion matrix of Carcinoma cell lines, the dataset was trained and tested, and the DT and RF algorithms were, again, implemented. For the training set, the DT correctly classified all instances of non-toxic (0) data points, and misclassified only 1 of toxic (1). This means that 259 out of 260 toxic rows were correctly predicted. In the table, the next horizontal cell, i.e., Actual 0 (toxic) and Predicted 1 (non-toxic), shows the value 1. This means that only 1 out 260 toxic rows were predicted as non-toxic. The first cell of the second row, i.e., Actual 1 (non-toxic) and Predicted 0 (toxic) in the table, shows a the value 1. This means that only 1 out of 474 non-toxic data points were predicted as toxic. For the test set, DT correctly classified all non-toxic data points as 0 and toxic data points as 1. Similarly, for both the training and test sets, RF correctly classified all non-toxic data points as 0 and toxic data points as 1.

DT and RF correctly classified all non-toxic (0) and toxic (1) data points in carcinoma cell lines. In addition, the tree diagram represents the prediction of cell viability, where outliers are removed for precise prediction. Thus, the tree shows an important attribute in predicting cell viability.

**Figure 5:**
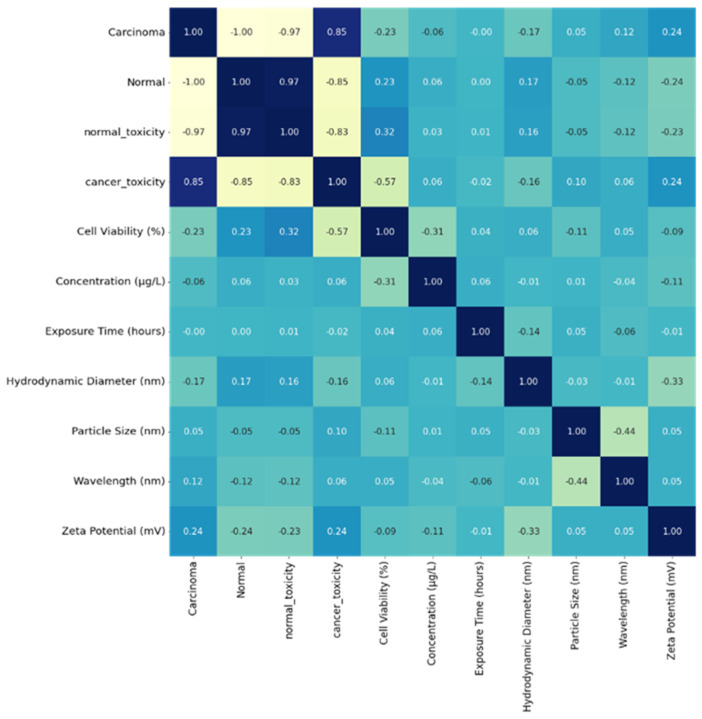
Heat map representing the density of the parameters leading to cytotoxicity of cell lines.

A heat map shown in Figure 5 was generated simultaneously, representing the correlation between the parameters on each axis.

The heat map ranges between −1 and +1—the estimated value is approximately zero, showing no linear relation between the two features. A correlation value near +1 indicates a strong positive correlation between the parameters, and -1 indicates a weaknegative correlation between the parameters, such that the value of one parameter increases and the other decreases. The diagonal values are 1, depicting an optimum correlation with each parameter correlating to itself.

Unsupervised learning is a subclass of machine learning. These models do not require labels for the input data or sample outputs. Each one searches for patterns and trends in the data. The used models run on unlabelled data after being trained on it. Clustering or cluster analysis is a machine learning technique that groups unlabelled datasets. The method follows dividing data points into groups such that each data point in a group is similar to other data points in the same group, and dissimilar to the data points in different groups. It is a collection of objects based on similarity and dissimilarity. The first step is randomly selecting several clusters represented by a variable ‘k.’ Next, each cluster is assigned a centroid, i.e., the centre of that cluster. It is essential to define the centroids as far off from each other as possible to reduce variation. After all of the centroids are specified, each data point is assigned to the cluster whose centroid is closest to these data. Once all of the data points are assigned to respective clusters, the centroid is again assigned for each cluster. Once again, all of the data points are rearranged in specific clusters based on their distance from the newly defined centroids. This process is repeated until the centroids stop moving from their positions.

All nine input features were selected to form individual cluster assignments to predict the expected outcome. The centre of a cluster could not be obtained after iterations as the data points seemed too varied and distinct. Expectation-maximization is a two-step procedure used by the algorithm’s core component. The expectation step locates the closest centroid for each data point. Then, in the maximizing stage, the mean of all points for each cluster is calculated, and the new centroid is set. Once the centroids converge or match the assignment from the previous iteration, the sum of the squared errors (SSE) is computed to assess the quality of the cluster assignments. SSE is calculated as the product of the squared distances between each point and its nearest centroid.

Given that this is a measure of error, k-means seeks to reduce this value. However, this value could neither be minimized nor converged for our developed dataset to form a centroid. Some significant inferences were understood through clustering analysis, and the figures are illustrated through scatter plots.

All graphs representing the clusters of the physical properties are compiled in a supplementary document S1 (Supplementary Figures S1–S10).

## 4. Materials and Methods

**Figure 6:**
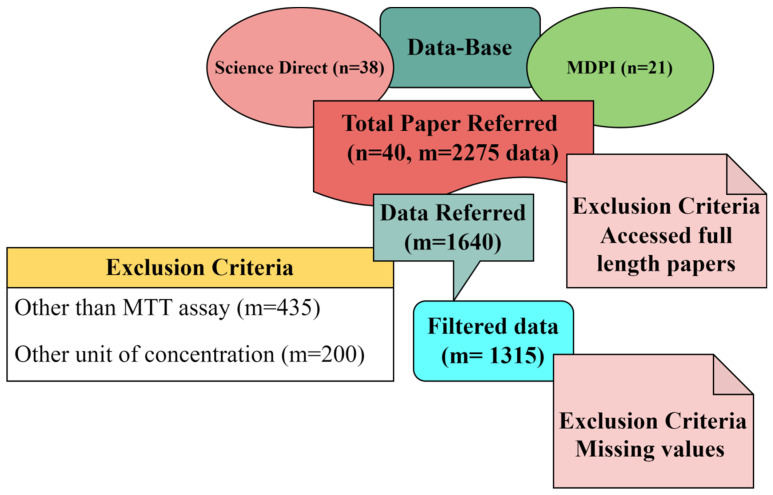
Block diagram representing the selection procedure of research articles.

Several efforts have been made in recent years to develop classic Quantitative Structure–Toxicity Relationship (QSTR) models. Unfortunately, their development is challenged by the inadequate number of toxicity-relevant physicochemical data in the dataset [21,25]. In contrast, the mature method called literature data mining (meta-analysis) is well known for establishing the relationships between the structural attributes and the toxic effects of numerous fabricated nanoparticles [22,26,27]. For data collection, the preferred keywords were “silver nanoparticles for cytotoxicity.” To avoid replication, we restricted ourselves to two different databases. One was ScienceDirect, and the other was MDPI. Also, we focused on the latest research outcomes in cytotoxicity and acquired the data from October 2017 to April 2022. We focused only on the latest research articles; therefore, we could not obtain sufficient data with only Ag-NPs as keywords for MTT assay. Thus, we increased the data by including chemically- and biologically-synthesized silver-modified nanoparticles. We found around 38 research articles from ScienceDirect and 21 from MDPI. Out of 59 articles, approximately 39 papers were accessed, and the 40th paper was our own previously reported article [24]. Finally, we collected 2275 datasets (cell viability and their corresponding dosages, i.e., concentrations of NPs). Of 2275 data sets, 435 were excluded because different assays were used to study the cytotoxicity other than the MTT assay. Two-hundred more data points were excluded, as the concentrations were measured in other forms rather than μg/mL. The outcome of the data mining was 1640 data points. Out of 1640 data points, 815 data points were of carcinoma cell lines and 825 data points were of normal cell lines. Further, 325 data points were discarded from normal cell lines due to more than 50% of the parameters missing out of the 9 inputs considered for the Machine Learning algorithm. Finally, 1315 filtered data points (500 normal cell lines and 815 carcinoma cell lines) from 40 articles, with the previously mentioned 9 different input features in determining cytotoxicity tested using MTT assay, were used. The schematic representation of the selection criteria of the datasets is shown in Figure 6.

A list of the research articles used to develop a dataset for building machine learning models has been provided in the Supplementary Document as Table S1.

We have recently reported cytotoxicity studies on Ag-NPs derived from two different routes. Turmeric, aloe vera, and turmeric mixed with aloe vera were used to obtain Ag-NPs. Among all of the synthesized samples, Ag-NPs derived from the turmeric extract were biocompatible with HEK 293 cells and non-toxic to the Drosophila model up to 250 µg/mL of concentration [24]. The cytotoxicity study was done on the HEK cell line, and the toxicity study was done on the Drosophila model [24]. The non-toxicity could be because of the shape, size, and better stability of NPs. The Ag-NPs were a medium for an anti-cancer study on the PC 12 carcinoma cell line. Biosynthesized turmeric-derived Ag-NPs were used to study the proliferative activity of PC 12 carcinoma cells using (3-(4,5-Dimethylthiazol-2-yl)-2,5-Diphenyltetrazolium Bromide) or MTT assay. In a 96-well plate, PC 12 cells were seeded and incubated overnight. The cells were treated with a turmeric-AgNPs plate in a dilution range from 0.1 to 100 g/mL. The treatment lasted 24, 48, 72, and 96 h. Following the indicated incubation time, 20 µL of MTT reagent was added to each well and incubated for 4 hours in a humidified 5% CO_2_ incubator at 37 °C. After 4 hours, each well received 100 µL of stock solution and was incubated for 1 hour to solubilize the formazan. Absorbance at 570 nm was recorded using a microplate reader (Bio Tek, Winooski, VT, USA). This report used DT, RF, and k-means clustering to understand the relation of key parameters with toxicity. The synthesized turmeric-derived Ag-NPs were used to study their cytotoxicity on carcinoma cell lines (PC 12) using MTT assay, which analyses the physical parameters that affect the cytotoxicity (as discussed in our previous study); exact classification and prediction were made with the help of DT and RF. Even k-means clustering indirectly predicted that the physical parameters which affect cell viability are interdependent and hence showed complex clusters for all of the parameters.

## 5. Conclusions

In our previously published work, various physical properties of Ag-T-NPs were studied. We reported that, more than other physical parameters, stability plays a vital role in the cytotoxicity (in-vitro test) of the NPs in normal cell line and in in-vivo toxicity. In continuation of our previous work, we have also analysed the optical property of Ag-T-NPs. Ag-T-NPshave been used to carry out cytotoxicity on carcinoma cell lines. Through Machine Learning, we tried to comprehend the relationship between toxicity and physical input features such as reducing agents, particle size, zeta potential, cell type (cancer/normal cell lines), hydrodynamic diameter, wavelength, morphology, exposure time, and exposure dosage. We carried out this study through two well-known supervised machine learning algorithms for regression analysis: Decision Tree (DT) and Random Forest (RF). The obtained test scores were compared with the DT and showed a perfect accuracy of 1 compared to RF. The obtained high value of R^2^ and low value of RMSE indicated that the prediction was accurate, suggesting that DT performed better than RF in predicting the toxicity parameter. The prediction was more precise and accurate, and best fit the dataset. The k-means clustering was used to analyze the relationship between different features; however, clear clusters were not formed. This was due to the relatively small dataset with varied features.

The close relationship that we have achieved between the experimental analysis results and the algorithm-based predictions shows the reliability of these algorithms. Hence, we suggest that these algorithms can be utilized as an add-on for optimizing and designing the synthesis of Ag-NPs for extended applications such as drug delivery and cancer treatments.

## Figures and Tables

**Table 1:** Represents the hyperparameters utilized for the algorithms.

Algorithm	Hyperparameters	Values (Carcinoma and Normal Cell Lines)
Random Forest (RF)	Number of Trees	10
	Number of attributes at each split	5
	Replicable Training	True
	Limit Depth of Individual Trees	15
	Don’t Split the subset smaller than	5
Decision Tree (DT)	Induce Binary Tree	True
	Minimum Numbere of Instances in leaves	2
	Don’t Split the subset smaller than	5
	Limit Maximum Tree Depth to	100

**Table 2:** Regression on Cytotoxicity associated with Carcinoma and Normal* cell lines.

Model	MSE	RMSE	MAE	R^2^
DT	160.86	12.68	8.80	0.84
RF	240.13	15.49	12.58	0.76
* DT	17.83	4.22	2.49	0.97
* RF	94.97	9.75	7.32	0.87

**Table 3:** Actual Values against Predicted Values.

	**Predicted Values**		
**Actual Values**		**Negative (0)**	**Positive (1)**
	Negative (0)	True Negative (TN)	False Negative (FN)
	Positive (1)	False Positive (FP)	True Positive (TP)

**Table 4:** Normal and Carcinoma cell lines Confusion Matrix.

Normal Cell Lines Confusion Matrix																			
DT										RF									
Train	Predict				Test	Predict				Train	Predict				Test	Predict			
		0	1				0	1				0	1				0	1	
Actual	0	279	0	279	Actual	0	31	0	31	Actual	0	279	0	279	Actual	0	31	0	31
	1	0	171	171		1	0	19	19		1	0	171	171		1	0	19	19
		279	171	450			31	19	50			279	171	450			31	19	50
**Carcinoma Cell Lines Confusion Matrix**																			
**DT**										**RF**									
**Train**	**Predict**				**Test**	**Predict**				**Train**	**Predict**				**Test**	**Predict**			
		**0**	**1**				**0**	**1**				**0**	**1**				**0**	**1**	
Actual	0	259	1	260	Actual	0	29	0	29	Actual	0	260	0	260	Actual	0	29	0	29
	1	1	473	474		1	0	52	52		1	0	474	474		1	0	52	52
		260	474	734			260	52	481			260	474	734			29	52	81

## Data Availability

Cited the reference papers that were utilized for developing a dataset for building the machine learning models in the methodology section.

## Supplementary Materials

The following supporting information can be downloaded at: https://www.mdpi.com/article/10.3390/ijms24044220/s1.
