# Supplementary material for: Meta-Analysis of Cytotoxicity Studies Using Machine Learning Models on Physical Properties of Plant Extract-Derived Silver Nanoparticles
Source: Int J Mol Sci. 2023 Feb 20;24(4):4220. doi: 10.3390/ijms24044220 (PMC9966579; doi:10.3390/ijms24044220)
Supplement: Supplementary file 1 [file ijms-24-04220-s001.zip › Supplementary ijms-2171650.pdf]

Supplementary Document 1

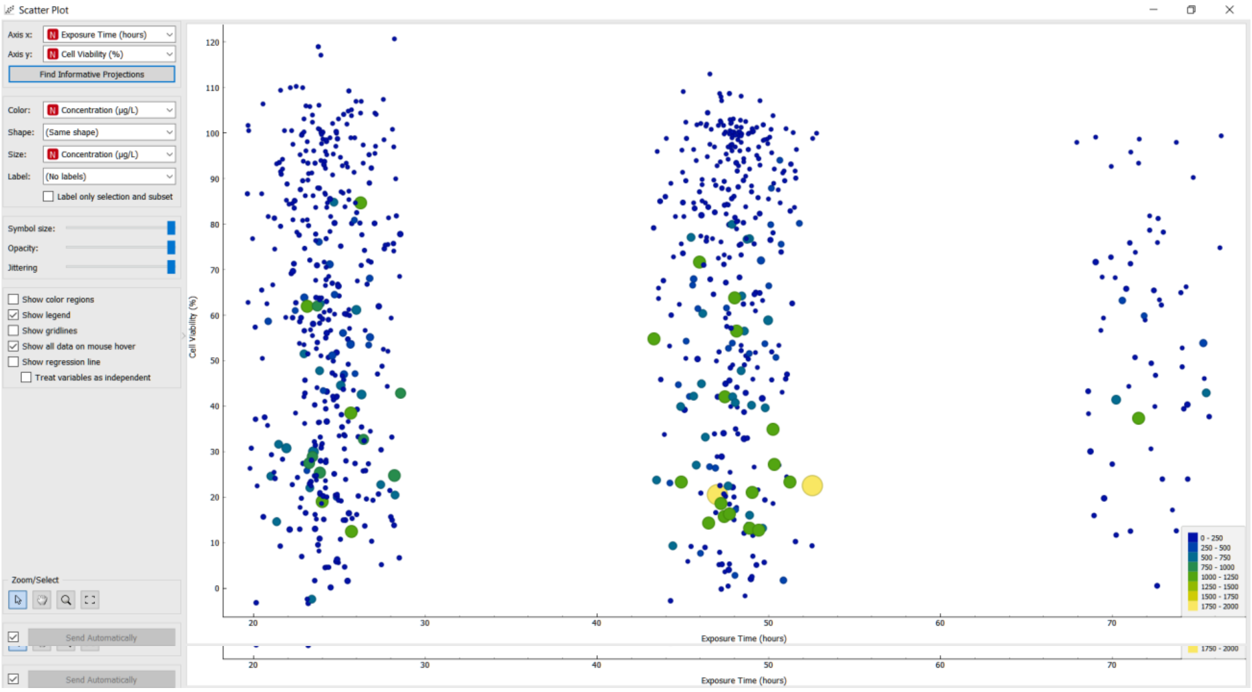

Figure S1. Graph representing cell viability vs exposure time respective to concentrations of nanoparticles for the carcinoma cell lines.

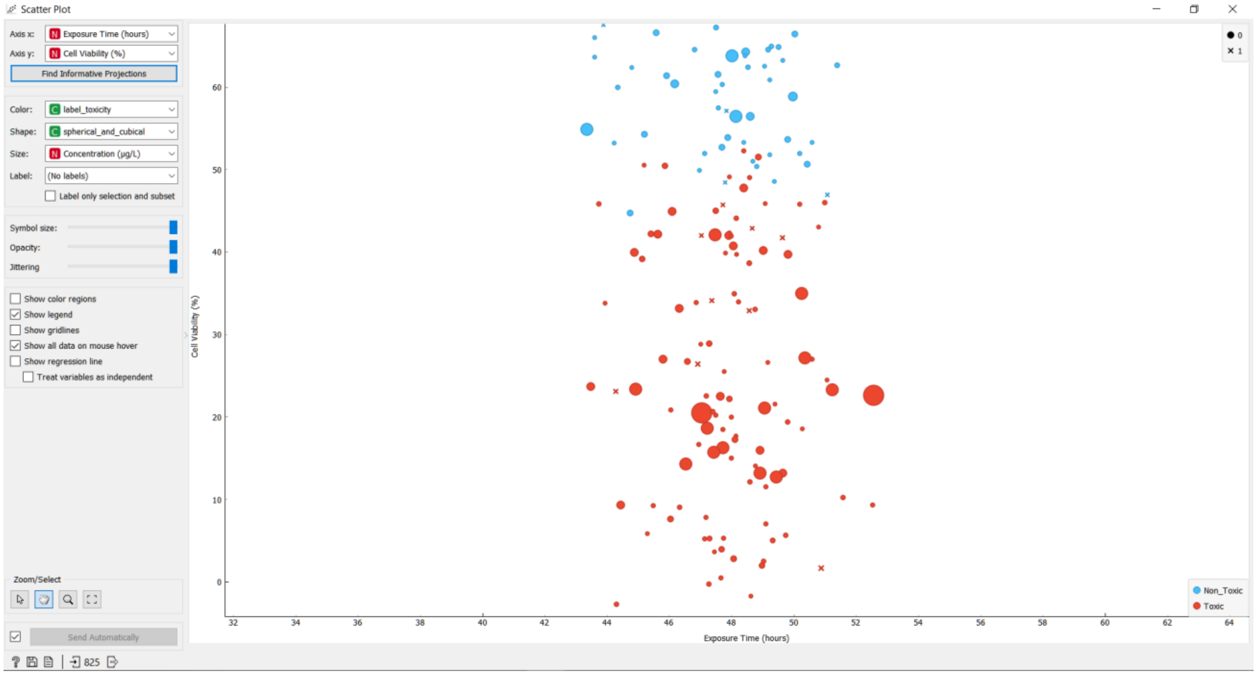

Figure S2. Graph representing cell viability vs exposure time respective to the morphology of nanoparticles for the carcinoma cell lines.

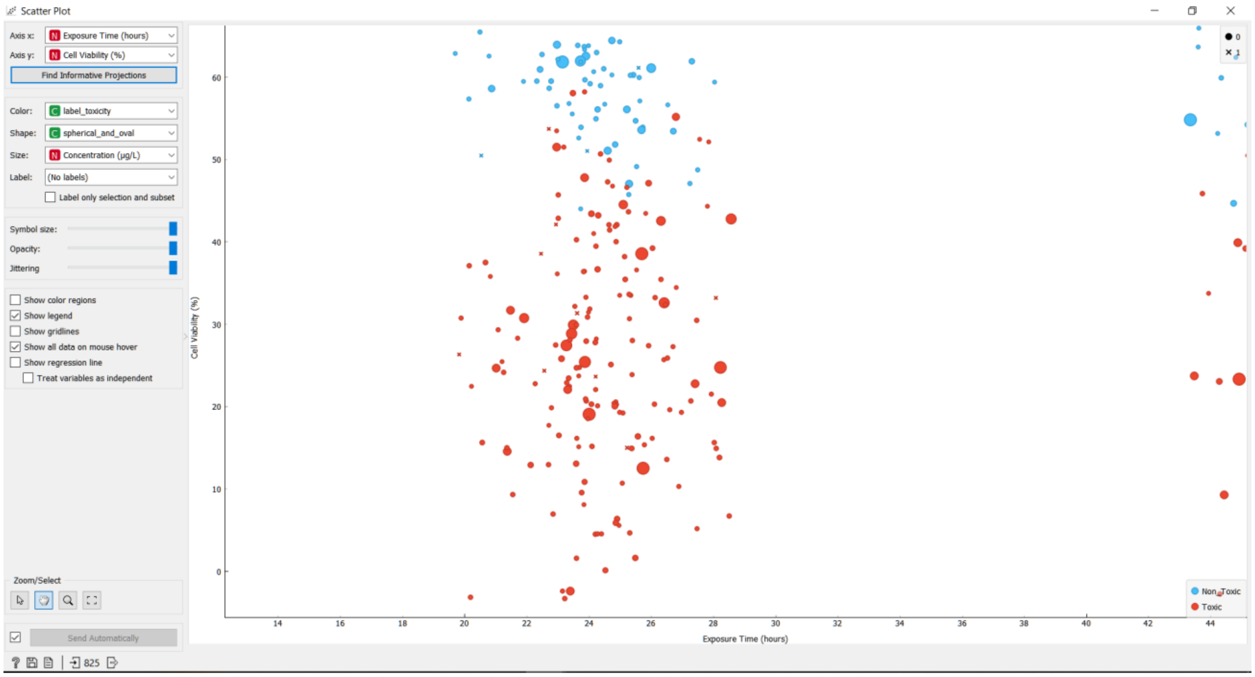

Figure S3. Graph representing cell viability vs exposure time respective to the morphology of nanoparticles for the carcinoma cell lines.

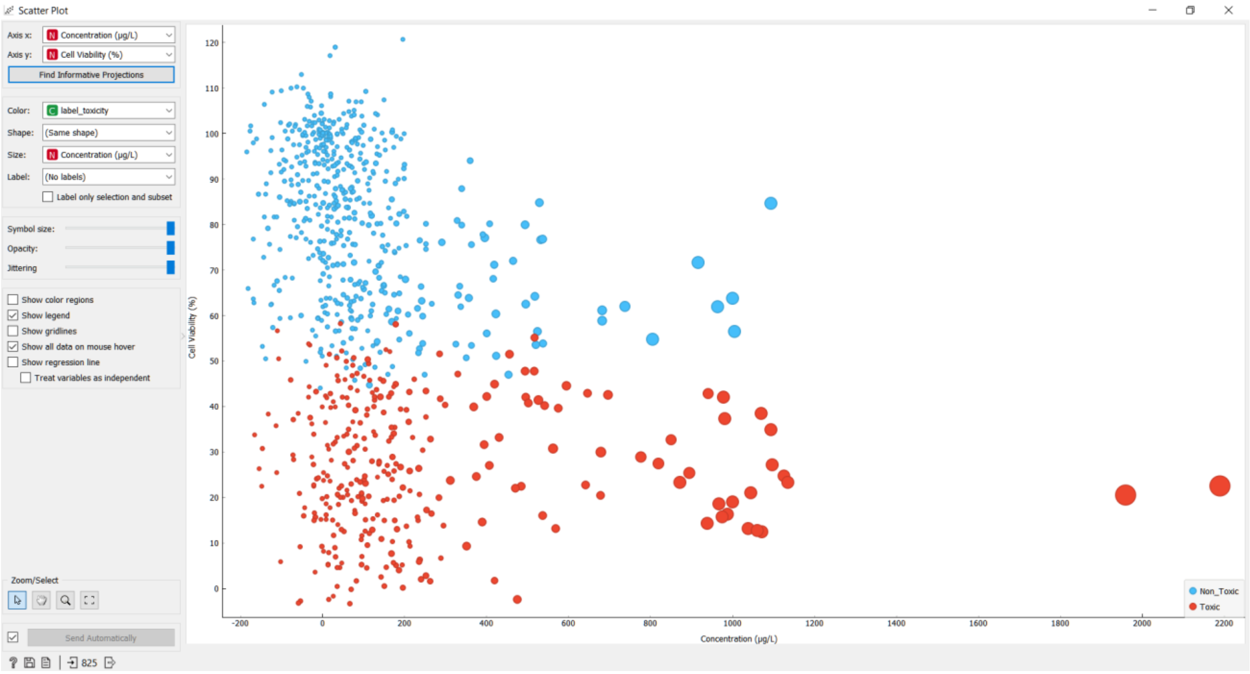

Figure S4. Graph representing cell viability vs concentrations of nanoparticles for the carcinoma cell lines.

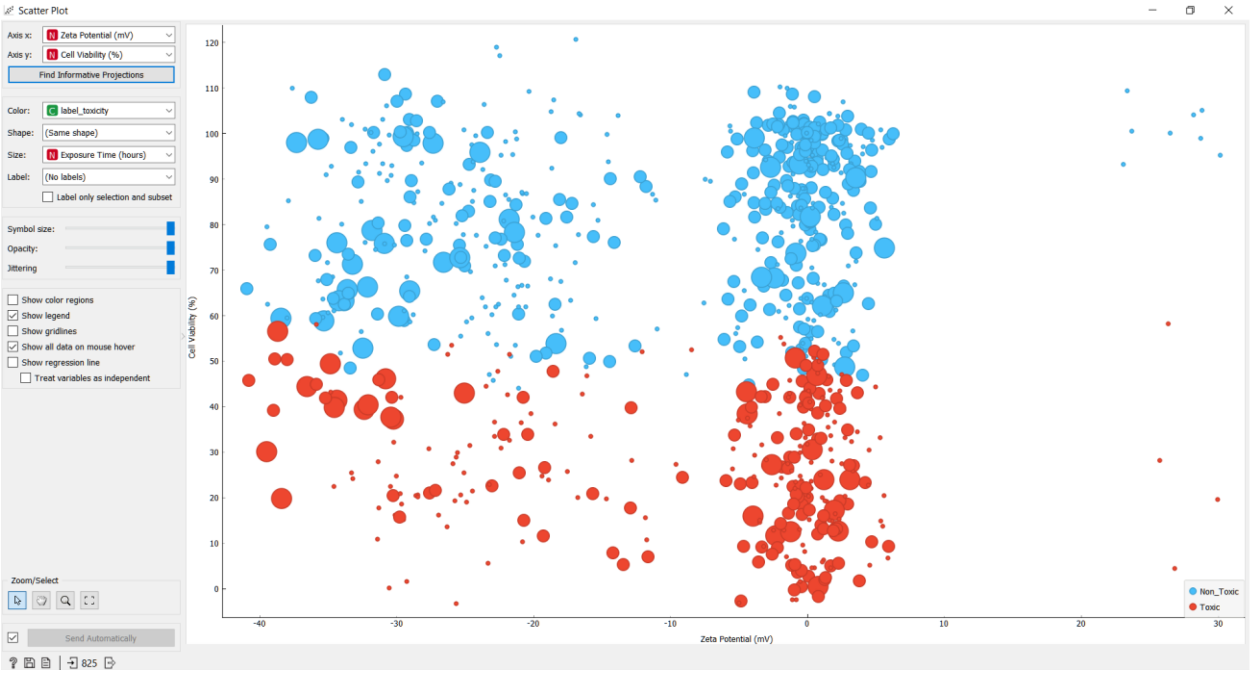

Figure S5. Graph representing cell viability vs zeta potential respective to exposure time of nanoparticles for the carcinoma cell lines.

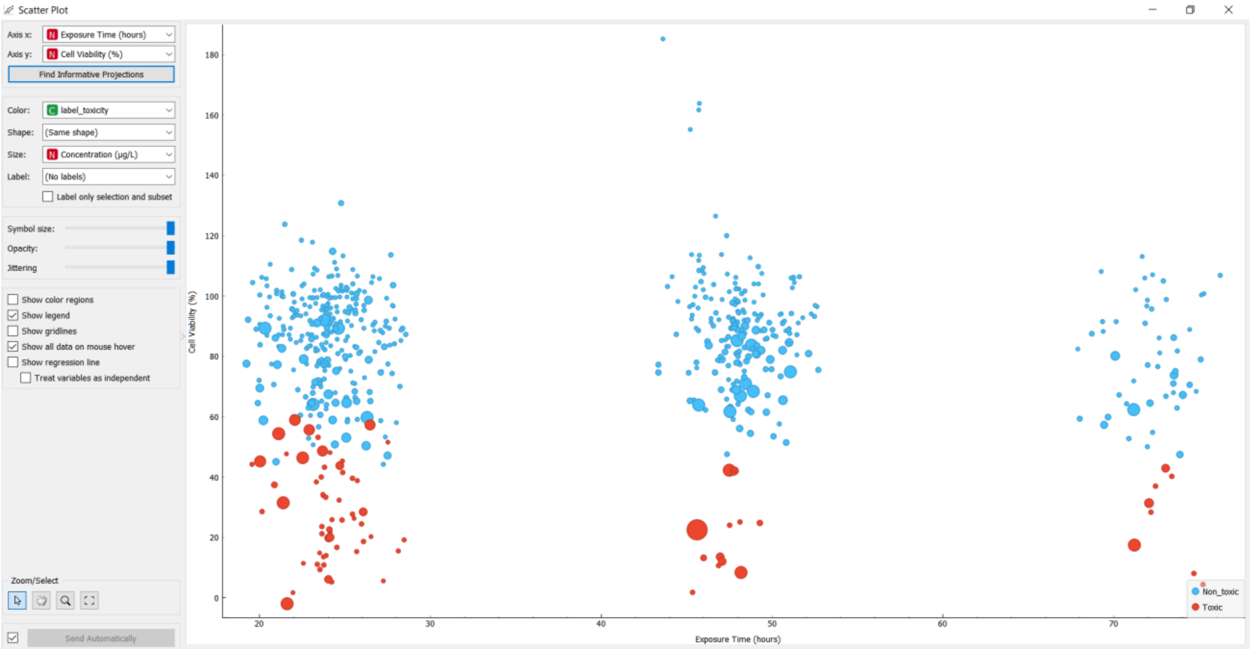

Figure S6. Graph representing cell viability vs exposure time respective to concentrations of nanoparticles for the normal cell lines.

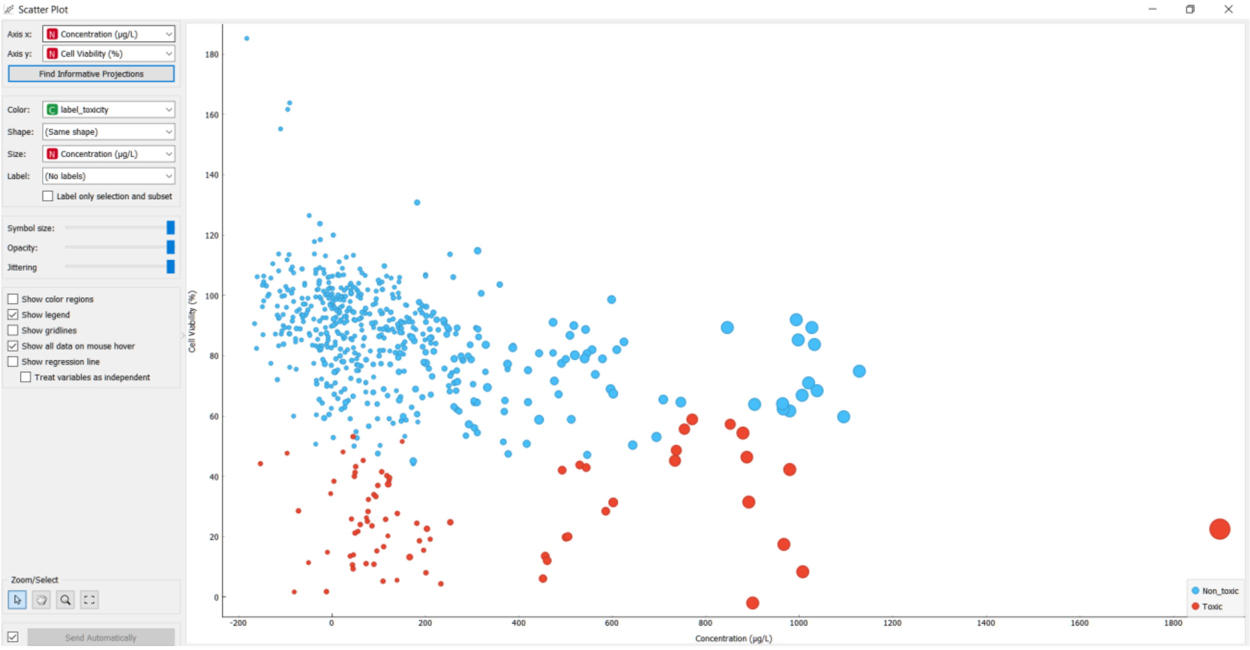

Figure S7. Graph representing cell viability vs concentrations of nanoparticles for the normal cell lines.

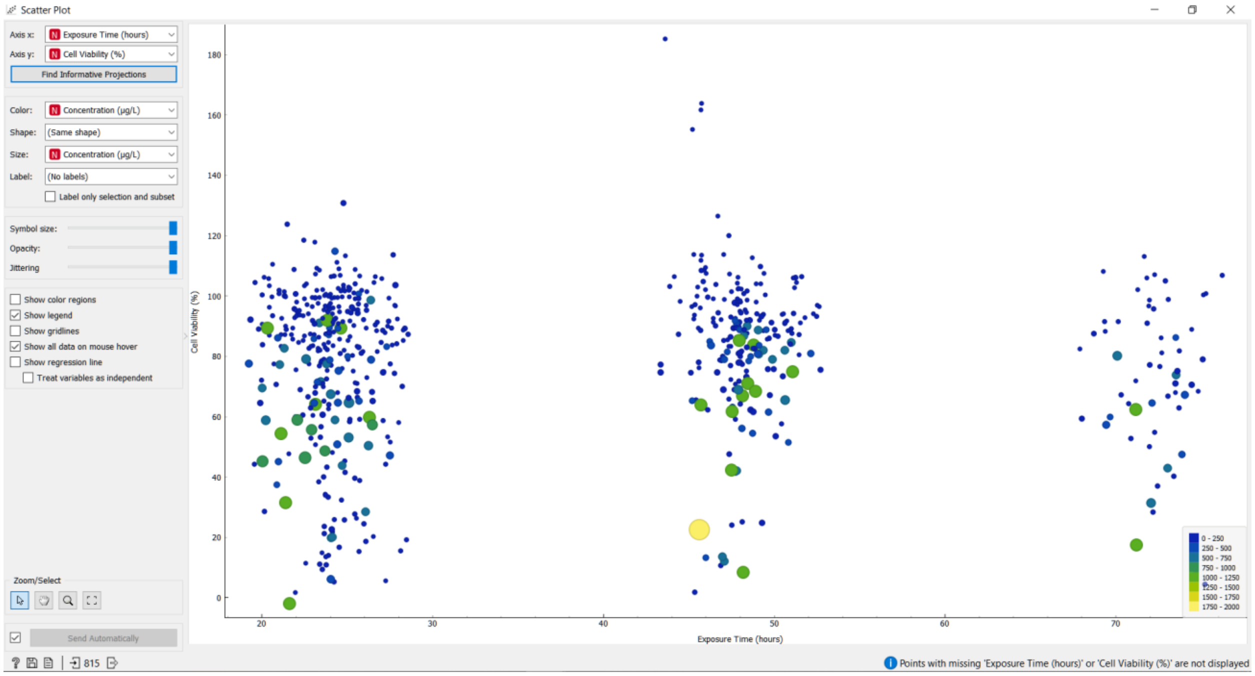

Figure S8. Graph representing cell viability vs exposure time respective to concentrations of nanoparticles for the normal cell lines.

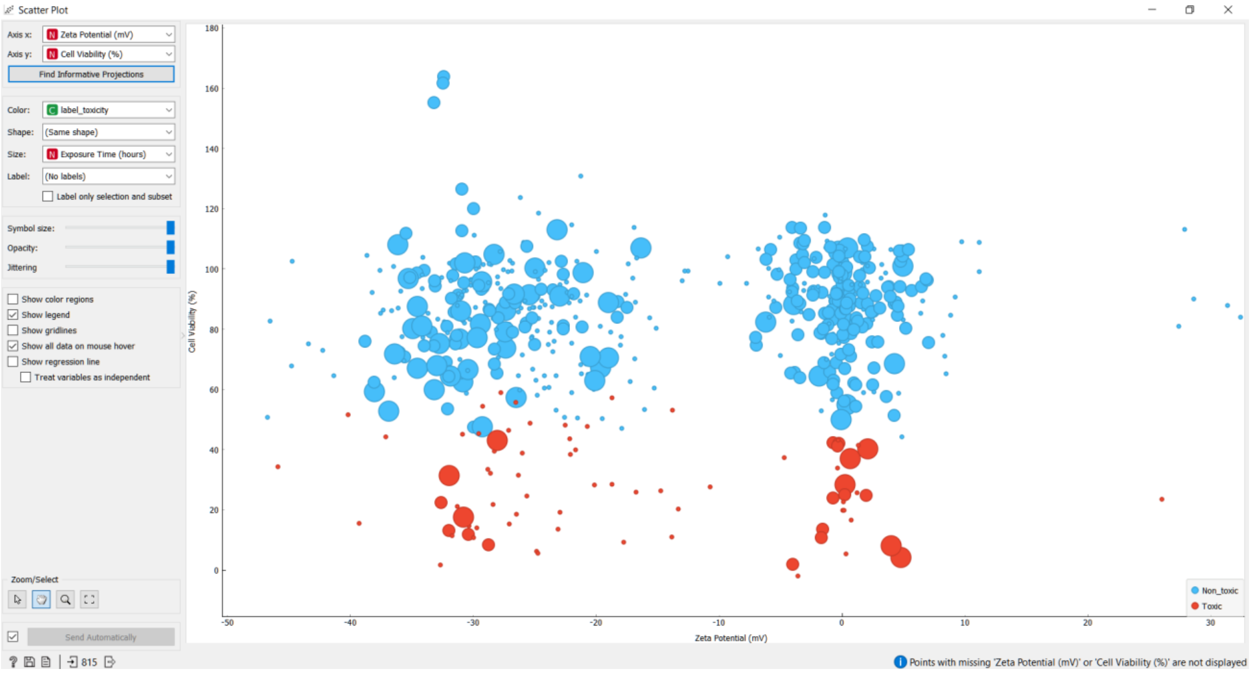

Figure S9. Graph representing cell viability vs zeta potential respective to exposure time of nanoparticles for the normal cell lines.

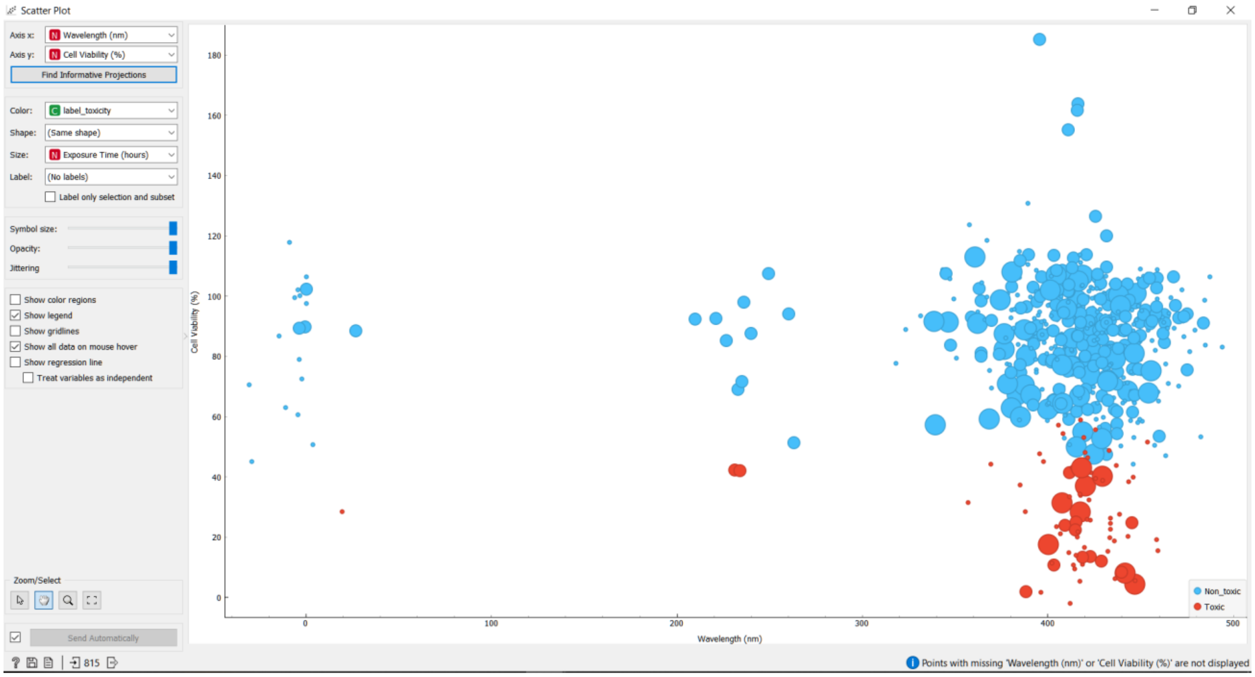

Figure S10. Graph representing cell viability vs wavelength respective toexposure time of nanoparticles for the normal cell lines.

Table S1. Research articles that were utilized for developing a dataset for building the machine learning models.

Research articles that were utilized for developing a dataset for building the machine learning models					
**Sr.No.**	**Author Name and Journal**	**Publication Year**	**Dataset**	**Entity Used**	**Results**
1	Vishnu Priya, et al., *Saudi Journal of Biological Sciences*	2021	Experimental	Animal L929 fibroblast	Confirms that the silver nanoparticles synthesized with Scutellariabarbata aqueous extract area potent wound-healing drug
2	Pallavi S.S., et al., *Saudi Journal of Biological Sciences*	2021	Experimental	Human A549	Evident that Streptomyces hirsutus strain SNPGA-8 AgNPs are potentially promising to be applied for biomedical uses.
3	Anushree Pandey, et al., *Materials Today: Proceedings*	2021	Experimental	Human MDA-MB-231	Encapsulated nanoparticle drug conjugate

					demonstrated a significant response against breast cancer cell lines
4	Mani M., et al., *Environmental Research*	2021	Experimental	Human MCF-7	MTT assay showed the anti-cancer activity of silver nanoparticles in a dose-dependent manner and is undoubtedly a potential agent for breast cancer
5	Gudikandula Krishna, et al., *Heliyon*	2021	Experimental	Human A549 and MCF-7	Prepared nanoparticle materials were found to be effectively potent against lung and breast cancer cell lines.
6	Yonghong Kong, et al., *Arabian Journal of Chemistry*	2021	Experimental	Human HUVEC, WiDr,SW1417, DLD-1, and LS-123	In cellular models, silver nanoparticles revealed significant anti-colorectal carcinoma activities against colorectal carcinoma cell lines. In vivo and clinical trials confirmed the same in humans
7	Yunjian Wang, et al., *Arabian Journal of Chemistry*	2021	Experimental	Human HUVEC, PANC-1,ASPC-1, DLD-1 and PaCa-2	Silver nanoparticles had very low cell viability and anti-human pancreatic cancer properties dose-dependently on the chosen cell lines
8	Azmat Ali Khan, et al., *Saudi Journal of Biological Sciences*	2021	Experimental	Human THP-1 and MDA-MB-231	AgNPs embodies intriguing anti-cancer potential against metastatic breast cancer cells.
9	Leeba Balan, et al., *Journal of Molecular Structure*	2021	Experimental	Human MCF-7	Synthesized nanoparticles showed a remarkable antimicrobial property at against the human pathogenic organisms used

10	Swarnendra Banerjee, et al., South African Journal of Botany	2021	Experimental	Human HEK-293	AgNPs may have the potency to combat cancer by manipulating apoptosis.
11	Pratik Das, et al., *Environmental Research*	2022	Experimental	Human	Phytochemicals attached to nanoparticle surface might be playing a crucial role to suppress the lethal effect of bare silver nanoparticles and also play a major role in their anti-cancer effect.
12	Gomathi A.C., et al., *Journal of Drug Delivery Science and Technology*	2019	Experimental	Human MCF-7	Silver Nanoparticles from Tamarindus indica fruit shell extract may act as a potential therapeutic agent for human breast cancer treatment
13	Mohammad Oveset, et al., *Saudi Journal of Biological Sciences*	2021	Experimental	Human MDA-MB-231	Nanomaterials were biocompatible and demonstrated the potential anti-cancer activities against MDA MB-231 cells after 24-hour exposure
14	Ali Naghizadehet, et al., *Environmental Technology & Innovation*	2021	Experimental	Human AGS and HFF	Biogenic AgNPs-JCE have an efficiency to degrade rhodamine b and eriochrome black T dyes under UV and visible light irradiations
15	Naser Abbasi, et al., *Arabian Journal of Chemistry*	2021	Experimental	Human HUVEC and MCF-7	The synthesized silver nanoparticles had excellent cell viability on the HUVECs line and indicated this method was non-toxic
16	Ozlem Kaplan, et al., *Journal of Drug Delivery Science and Technology*	2021	Experimental	Human MCF-7, HT-29 and HUH-7	Obtained results demonstrated that synthesized AgNPs had an average size diameter of <100 nm

					and have strong anti-cancer, antibacterial, antifungal and wound healing potentials.
17	Anam Rana Gul, et al., *Chemical Engineering Journal*	2020	Experimental	Animal SCC-7, RAW-264.7and Human HEK-293	The biological functionality and biocompatibility of a common agriculture waste based AgNPs, suggest their promising role as a potential drug carrier in the field of therapeutics
18	Krishanu Ghosal, et al., I*nternational Journal of Biological Macromolecules*	2020	Experimental	Animal OP-9 and Human HeLa, MCF-7	Anti-cancer activity of AgNPs is strongly related with the generation of intracellular ROS leading to apoptosis
19	M. Govindappa, et al., *Journal of Drug Delivery Science and Technology*	2020	Experimental	Animal L929 fibroblast, Human HeLa, MCF-7	Pomegranate fruit fleshy pericarp acts as natural agent to synthesize AgNPs, which may gain attention to treat P. aeruginosa infection
20	Sneha S. Rao, et al., *International Journal of Biological Macromolecules*	2020	Experimental	Animal NIH-3T3	Oxidative stress and intracellular protein leakage were observed due to AgNP interaction with the cell bringing about bactericidal effect.
21	Himabindu Padinjarathil, et al., *International Journal of Biological Macromolecules*	2018	Experimental	Human-HepG-2, A549 and HCT-116, Animal 373-L1	Nanoparticles displayed selective Cytotoxicity towards human adenocarcinoma, colorectal carcinoma, and hepatocellular carcinoma cells
22	Dongxiao Wang, et al., *Arabian Journal of Chemistry*	2022	Experimental	Human HUVEC, HT-29, HCT-116, HCT-8 and Ramos-	Anti-human colorectal carcinoma properties of Pectin/Ag NPs are related to their

				2G6.4C10	antioxidant effects.
23	Mostafa M. El-Sheekh, et al., *Environmental Nanotechnology, Monitoring & Management*	2021	Experimental	Human WISH, HeLa and CaCO-2	Low concentrations of silver oxide nanoparticles showed the safety limit effect against HeLa and CaCo-2, and no hazard on WISH cells
24	Shahnaz Majeed, et al., *Sustainable Chemistry and Pharmacy*	2019	Experimental	Human MCF-7, MG-63 and 3T3	Nanoparticles showed anti-cancer activity against MCF-7 and MG-63 cell lines and less toxicity towards normal human fibroblast 3T3 cells
25	Arul Kumar Murugesan, et al., *Environmental Nanotechnology, Monitoring & Management*	2021	Experimental	Human A549	AgNPs had potent anti-cancer activity against human lung cancer cell line (A549) by decreasing cell proliferation and inducing apoptosis
26	Nur Asna Binti Azhar, et al., *Toxicology in Vitro*	2020	Experimental	Human HePG-2	AgNPs have produced cytotoxic effects on HepG2 cells and have potential to be used as an anti-cancer treatment, for hepatocellular carcinoma
27	Jayashree Shanmugam, et al., *Nanomaterials*	2022	Experimental	Human MCF-7	Reduction in the feasibility of cancer cells was established via MTT assay, which suggests potential biomedical applications
28	Arpitha Badarinath Mahajanakatti, et al., *Molecules*	2022	Experimental	Human MCF-7	Based on the MTT assay result, it is summarized that the nanoconjugates possessed potential anti-cancer activity
29	Muthuraj Rudrappa, et al., *Nanomaterials*	2022	Experimental	Human U118MG	Developed an eco-friendly AgNPs synthesis using P. alba

					leaf extract with potential cytotoxic and antibacterial capacity
30	Abdulaziz A. Al-Khedhairy and Rizwan Wahab, *Metals*	2022	Experimental	Human HePG-2 and MCF-7	AgNPs induced Cytotoxicity and apoptosis via caspase pathways in cancer cells
31	Femi Olawale, et al., *Nanomaterials*	2021	Experimental	Human HEK-293	The NPs synthesized demonstrated significant free radical scavenging activity and a dose-dependent cytotoxic and genotoxic effect.
32	Surbhi Shinde, et al., *Applied Sciences*	2021	Experimental	Animal ATCC CCL-81	Silver and its bioconjugates from Prunus cerasus leaf extracts are noncytotoxic to produce antimicrobial compounds with low Cytotoxicity
33	Kaushik Kumar Bharadwaj, et al., *Nanomaterials*	2021	Experimental	Human U87-MG	AgNPs exhibited significant dose-dependent anti-cancer activity on U87-MG (human primary glioblastoma) cells
34	Mohd Shahnawaz Khan, et al., *Pharmaceutics*	2021	Experimental	Human MCF-7, HCT-116	Observed cell migration inhibiting potential of AgNPs in a concentration-dependent manner in MCF-7 cell lines
35	Magdalena Wypij, et al., *Molecules*	2020	Experimental	Human MCF-7, RAW-264.7	AgNPs displayed a high cytotoxic effect against cancer cells, their functionalization lead to their use in targeted cancer therapy
36	Hanan M. Al-Yousef, et al., *Processes*	2020	Experimental	Human HePG-2 and MCF-7	Ag NPs demonstrated considerable anti-cancer activity against the studied cell lines

37	Montserrat Lopez-Carrizale, et al., *Antibiotics*	2018	Experimental	Human fibroblast	Infections due to multidrug resistant microorganisms could be treated by the use of a synergistic combination of antimicrobial drugs and AgNPs
38	Fozia Shaheen, et al., *Nanomaterials*	2017	Experimental	Human MCF-7	Ag exhibited significant cytotoxic differences after the laser exposure and revealed apoptotic activity against a breast cancer cell line
39	Adewale O. Fadaka, et al., *International Journal of Molecular Sciences*	2022	Experimental	Human KMST-6, HT-29 and CaCO-2	Selective and reduced Cytotoxicity demonstrated by GAC-AgNPs toward colon cancer cells with the surface,thecomposition can be used to control the biodistribution, uptake, and efficacy of AgNPs.
40	Desai A.S., et al., *Journal of Functional Biomaterials*	2022	Experimental	Human HEK 293	Ag-NPs derived from the turmeric extract were biocompatible with HEK 293 cell lines
